# Zooming in on style: Exploring style perception using details of paintings

**DOI:** 10.1167/jov.23.6.2

**Published:** 2023-06-02

**Authors:** Yuguang Zhao, Jeroen Stumpel, Huib de Ridder, Maarten W. A. Wijntjes

**Affiliations:** 1 Perceptual Intelligence Lab, Faculty of Industrial Design Engineering, Delft University of Technology, Delft, The Netherlands; 2 Department of History and Art History, Utrecht University, Utrecht, The Netherlands

**Keywords:** style perception, multidimensional scaling, art history, property estimation

## Abstract

Most studies on the perception of style have used whole scenes/entire paintings; in our study, we isolated a single motif (an apple) to reduce or even eliminate the influence of composition, iconography, and other contextual information. In this article, we empirically address two fundamental questions of the existence (Experiment 1) and description (Experiment 2) of style. We chose 48 cut-outs of mostly Western European paintings (15th to 21st century) that showed apples. In Experiment 1, 415 unique participants completed online triplet similarity tasks. Multidimensional scaling (MDS) reached a nonrandom three-dimensional (3D) embedding, showing that participants are able to judge stylistic differences in a systematic way. We also found a strong correlation between creation year and embedding, both a linear correlation with Dimension 2, and a rotational correlation in the first two dimensions. To interpret the embedding further, in Experiment 2, we fitted three color statistics and nine attribute ratings (glossiness, three-dimensionality, convincingness, brush coarseness, etc.) to the 3D perceptual style space. Results showed that Dimension 1 is associated with spatial attributes (Smoothness, Brushstroke coarseness) and Convincingness, Dimension 2 is related to Hue, and Dimension 3 is related to Chroma. The results suggest that texture and color are two important variables for style perception. By isolating the motifs, we could exclude higher levels of information such as composition and context. Interestingly, the results reinforce previous findings using whole scenes, suggesting that style can already be perceived in sometimes very small fragments of paintings.

## Introduction

In his book *Principles of Art History*, Heinrich Wölfflin referred to an anecdote in which four German painters from the Romantic period tried to paint a particular scenery all “firmly resolved not to deviate from nature by a hair’s breadth” (Wölfflin, 2012). The resulting landscapes, however, differed considerably in style. Wöllflin ascribed this fact to differences in personality and vision of the artists. He also remarked that despite the differences, we would easily see the similarities between them and recognize them as products of a particular period: the first half of the 19th century. For Wölfflin, such collective differences between the pictorial production of different periods were ultimately rooted in differences in artistic vision or perception. To capture the differences between 16th- and 17th-century painters, Wölfflin came up with five visual principles: (1) linear versus painterly, (2) closed versus open form, (3) planar versus recessional, (4) multiplicity versus unity, and (5) absolute versus relative clarity. Despite their widespread use both within and beyond art history, such as in perception research (Goude & Derefeldt, 1981; O’Hare, 1979) and computer vision (Cetinic, Lipic, & Grgic, 2020; Elgammal, Liu, Kim, Elhoseiny, & Mazzone, 2018), it can be seen that these principles have their limitations and are specifically conceived to model the contrast between Renaissance and Baroque art.

To understand the matter of style, we need a broader definition that is both testable and can generate novel insights. Gombrich (1968) seems to offer this broader definition:

Style is a distinctive, and therefore recognizable, way in which an act is performed or an artifact made.

This is clearly a general description but at the same time specifically emphasizes the role of the beholder (“recognizable”). If there are no differences to be perceived, there is no style. This fundamental aspect of style (its existence) precedes descriptions or models of style such as those of Wölfflin. In this article, we empirically address these two fundamental questions of the existence (Experiment 1) and description (Experiment 2) of style in the context of visual perception.

###  

#### Style measurements

To empirically investigate the perception of style, one ideally refrains from any explicit terminology. A disadvantage of a Wölfflinian approach is the top-down usage of terms describing style differences, instead of a bottom-up approach that does not make use of such terms. The invention of multidimensional scaling (MDS) methods (see Mead, 1992, for a review) offered such an opportunity: Instead of relying on explicit adjectives, attributes, or descriptions, the MDS approach only relies on perceived differences (or “distances”), from which a space is constructed. This space is a low-dimensional representation of the theoretical high-dimensional space where each element would have its own dimension. This representation can be concisely referred to as “embedding” and sometimes, when appropriate, as “perceptual space.” Indeed, after substantial methodological progress was made in the 1960s (e.g., Shepard, 1962; Kruskal, 1964b), this approach became popular in style perception studies. Berlyne and Ogilvie (1974), for example, conducted a series of similarity judgments and attribute rating experiments on 52 paintings covering 14th- to mid-20th-century (Western) art. Observers were instructed to indicate “how similar or different the two pictures of each pair were” using a 7-point scale. The authors concluded that a three-dimensional (3D) space would be the most reasonable solution to explain their data, the first dimension being aligned with creation year. Interestingly, the authors had difficulty explaining the second and third dimension, and only very tentatively suggested an influence of line and surface quality. Importantly, they found reasonable interrater reliability, meaning that observers agreed quite well on perceived style. Referring back to Gombrich’s definition, the study by Berlyne and Ogilvie (1974) showed enough “distinctiveness” between the painting styles as demonstrated by interrater reliability and a style space of three dimensions with reasonable stress value (<0.2).

In addition to accessing perceptual spaces and interpreting them by means of explicit attribute ratings, similarity judgments data have also been used for classification schemes (Graham, Hughes, Leder, & Rockmore, 2012). Here, the similarity data can be utilized for identifying latent stylistic dimensions in an unsupervised model or for training classification models in a supervised manner (Hughes, Graham, Jacobsen, & Rockmore, 2011).

Other studies focus more on feature statistics, such as color histogram statistics (Rao, Srihari, & Zhang, 1999) or pixel information at the level of the brushstroke. Sablatnig, Kammerer, and Zolda (1998), for example, used a combination of face recognition and brushstroke analysis to classify paintings into different categories. However, it can often be unclear whether the algorithms are measuring what is represented (i.e., depicted scenes) or the medium (i.e., paint on the canvas). We will come back to this issue in the General discussion.

#### Style descriptions

Various attempts have been made to quantify which visual features describe style. For example, Berlyne and Ogilvie (1974) asked observers in further experiments to rate the paintings on various affective, descriptive, artistic, and stylistic scales. Especially interesting were the four scales of texture, lines, colors, and shapes. These scales are somewhat related to Wölfflin’s principles. They were mostly significantly describing the style space. Marković and Radonjić (2008) investigated the role of implicit and explicit features in style perception. In their terminology, implicit refers mostly to subjective impressions such as aesthetic and affective judgments while explicit refers to more “objective” features such as form, color, and space. Interestingly, they found that the MDS configurations of 24 paintings could mostly be explained by explicit features. The general approach of using attribute ratings to explain stylistic differences in paintings was also used in other studies. O’Hare (1976) used a mixture of implicit (e.g., like–dislike, interesting–uninteresting, peacefulness–disturbed) and explicit (e.g., dark–bright, soft–sharp, few–many colors) features. He found significant correlations between the first MDS dimension and “realism” and between the second MDS dimension and “clarity” and “symmetry.” These findings were rather robust as a follow-up study confirmed (O’Hare, 1979).

While attribute ratings have been used to explain style embeddings, they have also been used to predict style categories: Ruth and Kolehmainen (1974) performed a factor analysis on attributes in relation to existing style labels. This approach thus assumes a fixed style structure that is different from the bottom-up approach of creating style embeddings like those using MDS. An interesting different approach to looking for style features is manipulating hypothesized features of style: Gardner (1974) altered texture and color by various image manipulations. Masking impaired style recognition, making it difficult to match artworks from the same artist.

From another perspective, Wallraven et al. (2009) proposed that humans use three levels of information for style perception: high-level background information: knowledge about specific historical events, knowledge about artists and art periods in general; mid-level content information: specific objects or scenes that are depicted, type of painting or subject (landscape painting, portrait, etc.); and low-level pictorial information: technique, thickness of brush strokes, type of painting material (oil, acrylic, etc.), color composition of the scene. They conducted three experiments to perform categorizing tasks. The results showed humans definitely need high-level information (old vs. new, perspective flat vs. open, etc.) to make style categorization judgments, although mid-level information (content, realistic vs. abstract, etc.) and low-level information (brush stroke, colors, etc.) were also used by some participants. Siefkes and Arielli (2018) also suggested that high-level information is important for style perception. It was argued that humans need knowledge about culture, history, or art categories to be able to perceive stylistic differences.

#### Computational studies

Besides behavioral research where the emphasis is on the human ability to perceive stylistic similarities of artworks, other studies have taken a computational approach. Graham, Friedenberg, Rockmore, and Field (2010), for example, related feature statistics to the axes of the MDS spaces reached from similarity judgments. Evidently, there have been major breakthroughs in so-called style transfer that started with Gatys, Ecker, and Bethge (2016), but this class of algorithms is not used to predict style differences and categories. Elgammal et al. (2018) used 20 style labels to train three deep convolutional neural networks (CNNs) on the WikiArt dataset. These CNNs achieved subspaces with fewer than 10 dimensions that explained 95% of the variance using principal component analysis (PCA) (Jolliffe, 2002), with the first two dimensions cumulatively explaining between 60% and 74%. Without having creation years or artists as input training data, the two-dimensional (2D) embeddings clearly showed a smooth temporal transition between styles, in a clockwise U-shape structure. The angular coordinates have a Pearson correlation coefficient of 0.69 with time, again suggesting creation year can be related to the style space. Furthermore, for 1,000 paintings, they collected art historians’ ratings of the Wölfflin principles and found correlations within the first five PCA dimensions. These ratings were then used by Cetinic et al. (2020) to do the reverse: train a network estimating the five principles, applying this to the original WikiArt dataset and look for patterns. They found an ascending trend of all five principles between the 15th and 17th centuries, corresponding to style change from Renaissance to Baroque.

#### Our contributions

The variety of paintings used in previous studies was often rather large. For example, the selection in Berlyne and Ogilvie (1974) contained still-lifes, portraits, biblical scenes, and abstract paintings. This makes it clear that style can refer to different levels (Wallraven et al., 2009) but that high-level background and mid-level content information were perhaps too dominant in their study, thus overruling potential low-level information. We hypothesize that the essence of style as defined by Gombrich will emerge more clearly when the subject matter is held constant, as in Wölfflin’s anecdote.

In an attempt to limit the influence of subject matter, O’Hare (1976) conducted an experiment with 12 landscape paintings. A 2D space was found where the realistic–unrealistic scale was connected to the first dimension and the clear–indefinite scale was connected to both first and second dimensions. Besides, we can observe an increase of creation year along the first dimension. Another attempt by Ruth and Kolehmainen (1974) used only paintings with similar content, “people surrounded by nature in each painting.” Yet in both artwork selections, the variety of subject matter is still rather large: People and landscapes can both vary tremendously in comparison to having artists depict exactly the same scene. Also, other elements in the scene (e.g., means of transport or dress) can provide time-related information, which corresponds to mid-level information proposed by Wallraven et al. (2009).

It is impossible to find a selection of paintings of the *exact* same subject matter, but we can isolate painting cut-outs of objects that are repeatedly depicted throughout art history. Ideally, the chosen motif does not undergo stylistic changes itself, which excludes humanmade objects such as clothing. The ideal motif is therefore something natural. A particular natural motif that is omnipresent throughout art history is an apple. Despite some texture and color differences, apples are relatively similar, especially concerning their shape and size. Isolating apples from their context from a wide variety of paintings and periods allows for an unprecedented control for subject matter and thus offers a unique window on the perception of style.

Second, attributes used to explain or create style embeddings often refer to the pictorial plane (e.g., brushstroke) and/or implicit features (e.g., aesthetic preference), usually ignoring features of pictorial representation. This may be due to the variation of subject matter, but it is undeniable that ways of depicting space and material are important aspects of style. Using square cut-outs of single objects will allow us to ask questions about object-specific properties (e.g., smoothness of depicted apple skin), regardless of the composition of the whole painting.

In most of the studies discussed above (e.g., Berlyne, & Ogilvie, 1974; O’Hare, 1976; Elgammal et al., 2018), creation year could be identified in the measurements on style differences and even related to the perceived realism of the painted scenes (O’Hare, 1976). But at the same time, it could be concluded that this is confined to paintings of whole scenes only. So, as a third contribution, we looked into whether the time a painting was created can also be revealed in observers’ style perceptions when both high-level background information and mid-level content information have been removed as much as possible.

Our fourth contribution is methodological. Many studies based on human judgments used pairwise similarity ratings. Both O’Hare (1976) and Linde (1975) have noted that pairwise similarity rating can be sensitive to individual differences: Scale range can vary considerably between observers and also depends on the preceding trials. Instead, we used triplet comparison to quantify style similarities. This has various potential advantages, one of which makes it possible to scale up the experiment across various participants. This would also allow for human judgments being used in computational scenarios. The computational style studies reviewed above are all based on existing style labels (e.g., from WikiArt) and not on perceived style differences. Although the number of paintings we investigated in the present study is still relatively small compared to computational studies, a methodological advancement is needed that could use human intelligence to form a lens through which artistic style is quantified, instead of the often-used computational lens.

In the present study, we address the issues outlined above. In the first experiment, we choose 48 apple cut-outs from paintings covering 1487 to 2017, reducing variability in content matter (Contributions 1 and 3). We used triplet judgments in combination with the method of Landmark MDS (De Silva, & Tenenbaum, 2004) where a subset of cut-outs (the so-called landmarks) is first used to create the initial MDS embedding and then used to fit the remaining nonlandmark cut-outs into this space. By doing so, we reduced number of trials dramatically (Contribution 4). In the second experiment, we performed multiple linear regression on a number of attributes, including some object-related features as opposed to the features about pictorial plane or implicit features (Contribution 2).

## Experiment 1: Similarity triplet ranking

### Method

#### Participants

The online experiments were conducted through Amazon Mechanical Turk (AMT), a crowdsourcing website for requesters (researchers in our case) to publish human intelligence tasks (HITs) online and hire crowd-workers (participants) to perform these HITs. In total, 415 unique participants completed Experiment 1 (98.8% were from North America, random sample).

All participants agreed with the informed consent before the actual experiment started and received compensation via AMT. The experiment was conducted in agreement with the Declaration of Helsinki and approved by the Human Research Ethics Committee of the Delft University of Technology. All data were collected anonymously.

#### Stimuli

**Figure 1.**
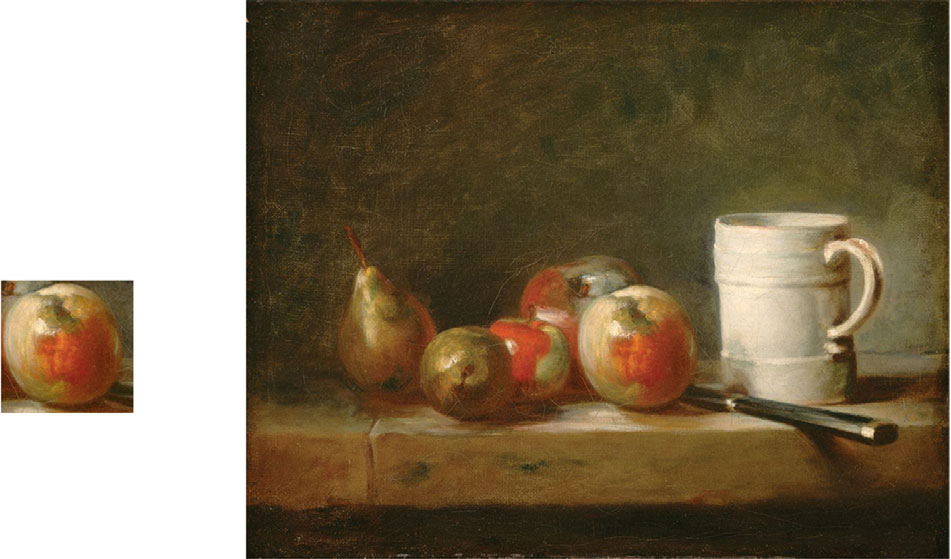
An example of a square cut-out of apple from an oil painting. Jean Siméon Chardin’s *Still Life With a White Mug* (1764), downloaded from the online repository of National Gallery of Art (nga.gov).

Forty-eight digital images of apple painting cut-outs were used as stimuli. All cut-outs were square cut-outs of high-resolution digital images retrieved from the “Materials in Painting Database” (Van Zuijlen, Lin, Bala, Pont, & Wijntjes, 2021) or online museum repositories. Most of them were oil paintings except for one or two that could have been painted in acrylic. Figure 1 shows an example of an original painting (on the right) and the square cut-out of an apple (on the left).

The creation years of the original paintings varied from 1487 to 2019. The selection covered artists from northern European countries (i.e., Netherlands, Germany) to southern European countries (i.e., Spain, Italy), and also paintings from France and North America.

Most square cut-out digital images have a resolution no less than 400 by 400 pixels and were set at 400 by 400 pixels in the online experiments. All images were embedded with an sRGB ICC color profile, so that browsers could display colors properly (Ashe, 2014).

#### Triplet comparison

To create a multidimensional embedding for a large set of images while distributing the judgments among many participants, we opted for a triplet comparison task over the pairwise similarity rating task. Apart from the before-mentioned advantages of this method, the disadvantage of using triplets instead of pairs is that the number of trials to create a (dis)similarity matrix increases enormously. For *n* stimuli, a pairwise method requires *n*(*n* − 1)/2 trials while a triplet method requires *n*(*n* − 1)(*n* − 2)/6 trials. In our case, with 48 stimuli, it would be 1,128 unique trials for the pairwise method versus 17,296 unique trials for the triplet method. To reduce the number of triplets to be evaluated, we used the method of Landmark MDS (LMDS).

#### LMDS

The original purpose of LMDS (De Silva & Tenenbaum, 2004) was to reduce computing power by using only a portion of the data to reach a final MDS solution without losing accuracy. In the current study, we used the method to reduce the number of trials. With LMDS, the first step is to select a subset of *l* stimuli as landmarks, either randomly or manually, and collect data and run a classical MDS analysis on those landmarks, that is, all *l*(*l* − 1)(*l* − 2)/6 triplets are being involved. The next step is to fit the remaining data points (*n* nonlandmarks) into the MDS space of landmarks, using distances between nonlandmarks and landmarks. For the first step, the lower half of an *l* × *l* full-distance matrix is required to run the MDS analysis. For the second step, only distances between nonlandmarks and landmarks are required. Thus, conventional MDS would require an (*l* + *n*) × (*l* + *n*) matrix, while for LMDS, only an (*l* + *n*) × *l* matrix is required to reach the final solution.

**Figure 2.**
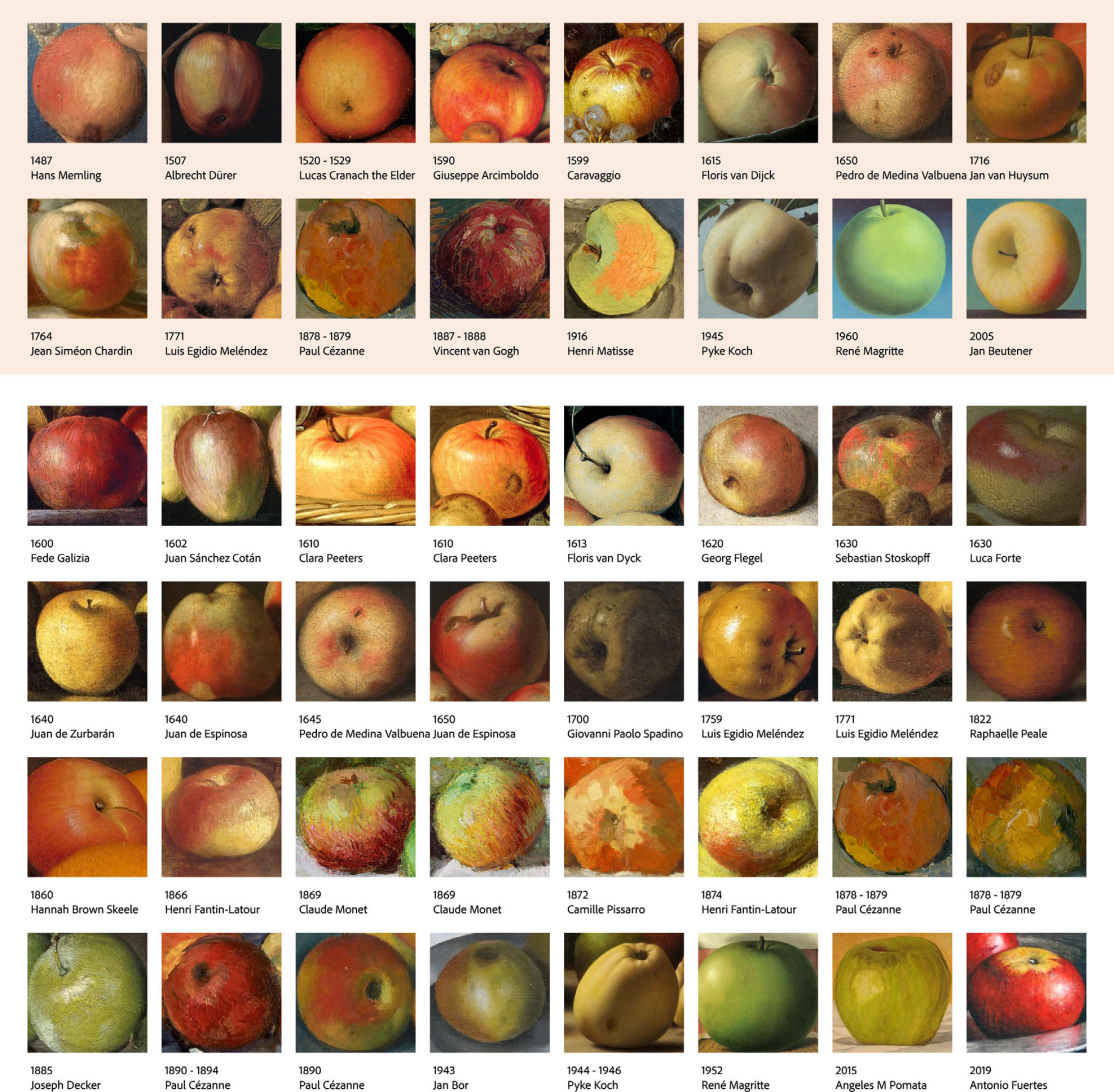
All 48 stimuli. The 16 landmarks are located in the upper part with light orange background, sorted by creation year. Thirty-two nonlandmarks are located in the lower part with white background, also sorted by creation year.

In the current study, 16 apple paintings were carefully chosen as landmarks, so that they represent various periods, and systematically distributed from north to south Europe (as shown in Figure 2, upper part with light orange background). We deliberately included two identical stimuli to verify this method. Paul Cézanne’s *Apples* (1778–1879) was used both as landmark and as nonlandmark. There were 16 landmarks (Ls) and 32 nonlandmarks (NLs). To generate the MDS space with the Ls, 16 × 15 × 14/6 = 560 triplets were needed. To fit the NLs in this space, each of the 32 NLs had to be paired with all unique pairs of Ls, that is, 32 × (16 × 15/2) = 3,840 triplets. In total, we presented 4,400 unique triplets, compared to 17,296 triplets without LMDS, a reduction of about 75%. We split trials into 40 experimental blocks, consisting of 110 trials with an average of 10 repetitions. At least 8 unique participants completed each of 40 subgroups (11 max, average = 10.38).

#### Procedure

Before the actual experiment, each participant would first read the consent form and instructions for the experiment. They could only proceed if they gave their consent by clicking “continue” after reading the consent form. Then they were presented the following instructions:

*STYLE: is the way things are done. People can have different driving styles, dancing styles etc. We are interested in painting styles. The aim of this experiment is to measure how humans perceive style differences. In paintings, style can show itself in various ways: the use of colors, shadows, lines, brushwork, light, shading, ordering, etc. But we preferably do not specify this exactly. In every trial, you will be shown three images of apples taken from larger paintings. You have to select the two that are most similar in style.*

**Figure 3.**
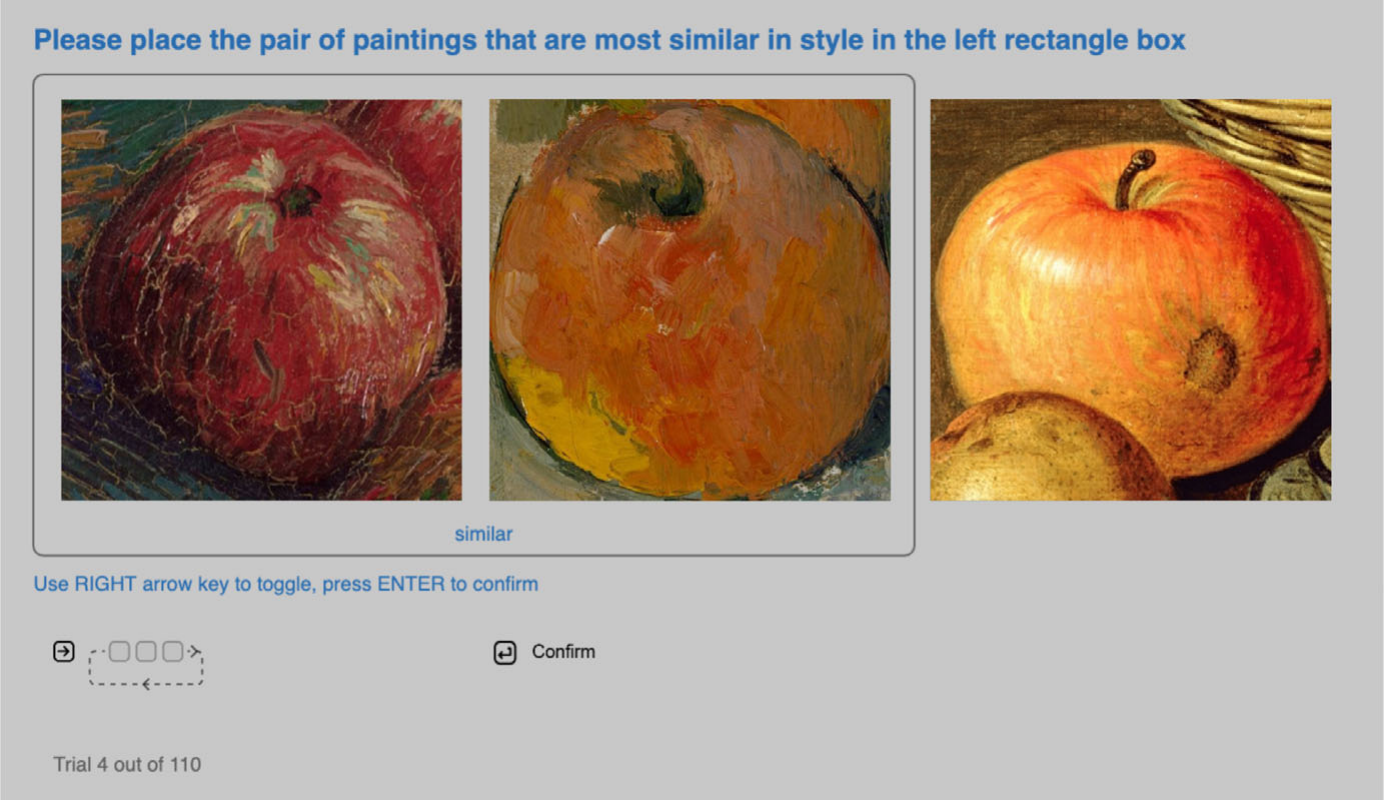
Interface for Experiment 1. In each trial, participants were presented with three square cut-outs of apples. Participants could use the *RIGHT arrow key* to toggle the order of the three cut-outs (as the left icon indicated), until the most similar pair in their opinion were in the rectangle box frame on the left. Then they could press *ENTER* to confirm and proceed to the next trial. On the bottom left, participants could see how many trials they still had to finish.

Then they went through five practice trials, to familiarize our interface and operation. In each trial, three stimuli were presented side by side (as shown in Figure 3). Participants were asked to place the most stylistically similar two stimuli in the rectangle box on the left. They could use the right arrow key on their keyboards to toggle the position of the three cut-outs, until the most similar pair was in the left rectangle box. They could press the enter key to confirm their choice and go to the next trial.

#### Data analysis

Before data analysis, we validated the data of individual participants on the basis of two criteria. We measured how much their answer deviated from the initial random setting (criterion at >15% change). Second, we used a minimum medium trial time of 1 second, a threshold used in a similar study one of the authors conducted before (Van Zuijlen, Pont, & Wijntjes, 2020). About 20% of the participants did not meet the selection criteria, and hence their data were removed for analysis, although these participants were reimbursed irrespective of this selection.

The data analysis consisted of two steps: First, a nonmetric MDS on the landmark stimuli was performed, and second, we fitted the other data to the LMDS configuration.

**MDS on landmarks**

The raw output of each participant consisted of 110 triplets. For landmark-only triplets, an output triplet (from left to right; see Figures 3A, B, C) meant the participant indicated the pair of Image A and Image B was the most similar pair. We created the (dis)similarity matrix using a frequency based method. For a pair A–B, the similarity score was calculated as follows (this is across the results from all participants encountering the pairs A–B): *s*/*t*, where *s* = amount of triplets where A and B were grouped together (the first two elements were AB or BA) and *t* = amount of triplets containing both A and B. The corresponding dissimilarity score was 1 − *s*/*t*.

With a dissimilarity matrix of 16 landmarks, nonmetric multidimensional scaling (NMDS) analysis was then performed with the metaMDS function from the vegan package (v2.5-6) in R (Oksanen et al., 2019). NMDS represents similarity data into a new configuration with the lowest possible dimensions. The best fit is achieved while the distances of landmarks are maintained as closely as possible. Compared to metric MDS, NMDS handles perceptual data better, since it arranges points to maximize rank-order correlation between real-world distance and ordination space distance (Shepard, 1962).

**Nonlandmarks into LMDS configuration**

We fitted nonlandmarks into the MDS space from the previous step using a brute-force procedure. The domain of the search extended twice the size spanned by the MDS locations and was split up in 60 evenly spaced sample points in each dimension. At each of these sampling points, fiducial triplet answers were generated on the basis of the MDS data and were compared to the participants’ triplets. Simulated triplets were thus compared with real triplets from participants’ answers. The cost function simply consisted of counting congruent triplets. To increase robustness, we took the average of the top 0.1% of this congruency score (227 in the current study).

### Results

**Figure 4.**
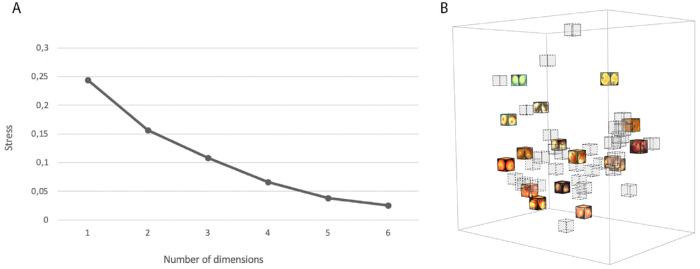
(A) Scree plot of MDS configuration of landmarks. (B) Three-dimensional configuration of only landmarks (gray dashed cubes are nonlandmarks fitted in the space later).

First, we determine the dimensionality of the landmark space by calculating the stress value as defined by Kruskal (1964a). The stress for one-dimensional to six-dimensional configurations is shown in Figure 4A. There is no obvious “elbow” shape, a commonly used criterion to determine dimensionality. Another common criterion is to choose the dimensions where the stress value is below 0.2 (Kruskal, 1964a). The first dimension that has a stress value below 0.2 is two. However, as the landmark set is only a subset of the image set, we decided to continue the analysis with three dimensions, as to not discard any potentially interesting patterns. As will be shown later, subsequent analyzes supported this choice.

Next, the 32 nonlandmark samples were fitted to the MDS space (Figure 4B, gray cubes). The cost function is the congruency between the actual answers and the answers constructed from the (to be fitted) configuration. We followed this brute-force fitting procedure for both the 2D and 3D configurations. The congruency values for both 2D and 3D solutions were well above chance level. There was a small but significant increase in congruency for the 3D embedding: (*t*(31) = −5.32, *p* < 0.001) reflecting an increase in congruency for 27 out of the 32 nonlandmark points. This supported the choice for using the 3D embedding for further analysis.

**Figure 5.**
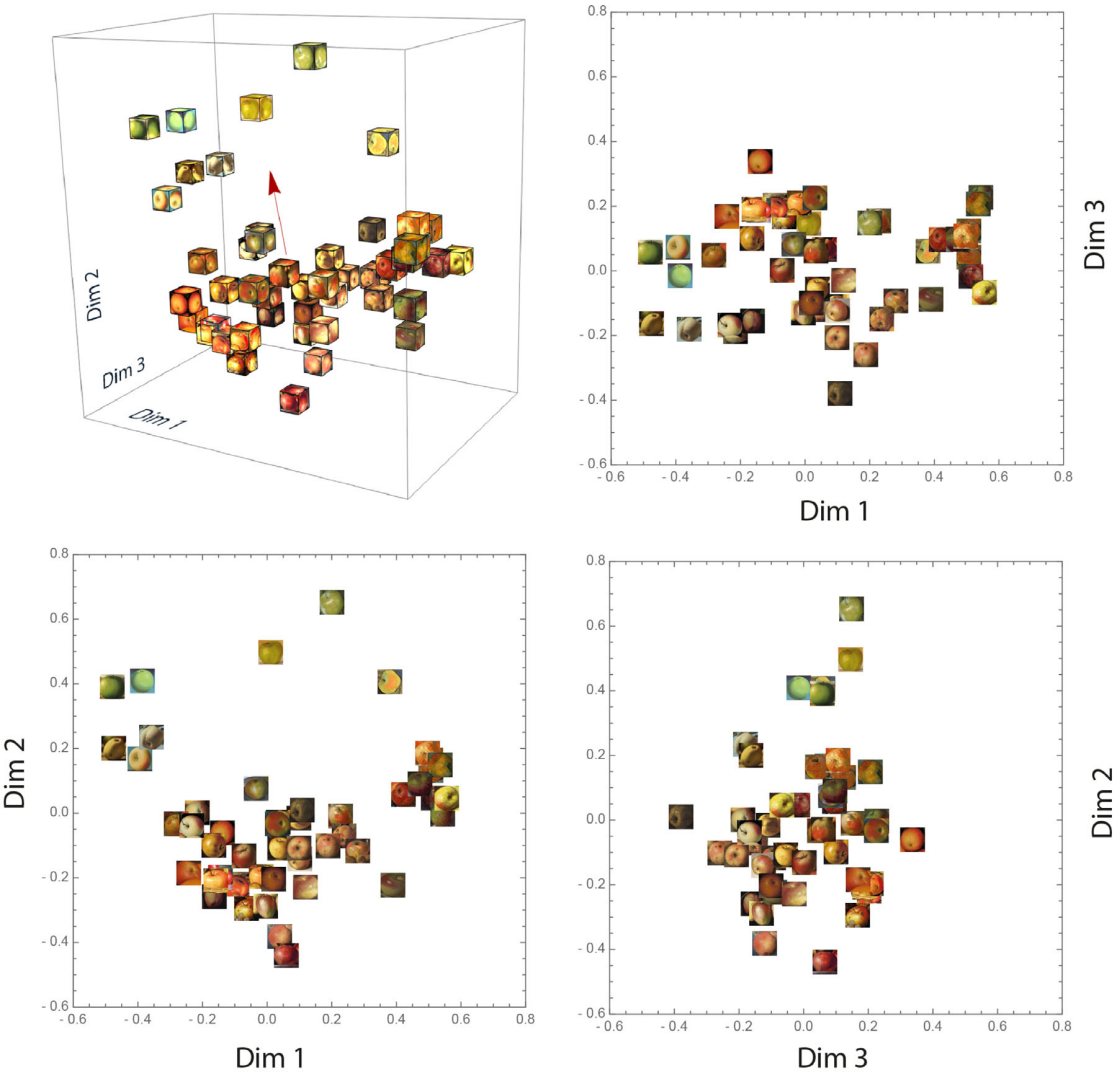
Three-dimensional space of style perception with 48 apple stimuli. Forty-eight boxes represented 48 stimuli. Each six faces of a box show the same apple image, so that it is visible from any viewing angle. The red arrow represents the vector of creation year fitted in the space.

Figure 5 shows the overall 3D embedding. As can be seen, the distribution is relatively homogeneous except for Dimension 2, where the distribution seems denser in the lower part. The two same stimuli for verification purposes locate very close to each other in the embedding, confirming the reliability of the landmark method. In addition, it seems that modern apples are on top of the space (along Dimension 2), while older apples are located lower. To further investigate this “historical dimension,” we performed a multiple linear regression for the creation year. With the set of coordinates and creation year for each stimulus as independent and dependent variables, respectively, the orientation of the vector indicates the direction that yielded the best regression, while the length indicates strength of the regression (*R*^2^). The red arrow in Figure 5 indicates this property vector.

**Figure 6.**
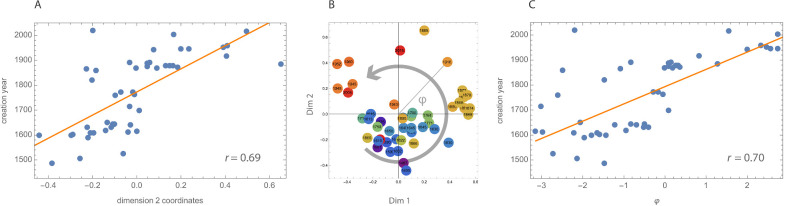
(A) Correlation between creation year and Dimension 2 in MDS space. (B) The first two dimensions with color-coded creation year and indication of a potential rotational correlation. (C) Correlation between creation year and rotation angle phi.

Statistical analysis revealed a significant overall fit ($R_{adjusted}^{2}=0.47$, *F*(3, 44) = 15.05, *p* < 0.001). Dimension 2 received the most weight distribution of the fit and is the only significantly associated dimension with creation year. Figure 6A shows the positive relation between creation year and Dimension 2 (*r* = 0.69, *p* < 0.001). To further explore the temporal aspect of the embedding, we plotted creation years in the first two dimensions in Figure 6B. This plot seems to suggest a rather clustered pattern with potentially a rotational correlation. Coincidentally, the data are distributed such that directly calculating the angle between the data points and the positive x-axis (Dimension 1) seemed to capture this trend (i.e., large negative angle and old creation year in lower left quadrant, intermediate angles and creation years in right quadrants, and large positive angle with new creation year in the upper left quadrant). This was confirmed by calculating the correlation between angle ϕ and creation year (*r* = 0.70, *p* < 0.001).

What can further be tentatively observed is that apples with coarse and visible brushstrokes are on the right side (along Dimension 1), while apples with fine and even invisible brushstrokes are on the left side. In addition, the greenish apples seem to be at the top of the distribution (along Dimension 2) with the reddish/yellowish ones at the bottom.

### Discussion

We found a nonrandom style embedding of a stimulus set where we held subject matter constant while using the LMDS approach. This suggests that even when high-level background information and mid-level content information have been removed by presenting a single object (apple) only, participants can still consistently perceive style differences. Apparently, there are object properties that make these judgments possible. This will be investigated further in Experiment 2 by assessing object-related attributes like smoothness and glossiness.

The dimensionality of the MDS analysis was based on 16 landmarks. As we mentioned in Results, it showed relatively low stress values for dimensions higher than 2 and there was no obvious elbow shape. So, additional criteria were needed. One of these criteria came from the fit of the nonlandmarks in the style space. For the majority of the nonlandmarks (27 out of 32), the data fitted better in the 3D embedding, which made us decide to continue our analysis with the 3D embedding, although stress levels suggested the 2D embedding to be already sufficient.

We fitted the creation year to the 3D embedding, and Dimension 2 resulted in a substantial correlation, *r* = 0.69 (Figure 6A). In addition, looking at the positions of the 48 cut-outs in Figure 5, the Dim1–Dim2 plane, a rotational pattern can be discerned, as demonstrated in Figure 6B. This rotational component can also be associated with creation year and yielded a somewhat higher correlation, *r* = 0.70 (Figure 6C). Interestingly, the half-hidden red circle in the third quadrant in Figure 6B represents a modern painting from 2019 amid a set of much older paintings. If we consider this painting as a continuation of the modern cluster from the second quadrant, in other words, if we add 360 degrees to the same data point in Figure 6C (the single point in the top left corner), the rotational correlation will even increase to *r* = 0.78. This rotational pattern is particularly interesting because a similar pattern was found by Elgammal et al. (2018), even though their embedding resulted from computational methods and very different experimental parameters. They used paintings of varying subject matter analyzed by a PCA on a CNN layer resulting from training on style labels, while we reached the embedding using human similarity judgment data. As our study and Elgammal et al. (2018) are so different, our finding strengthens the possibility that a cyclical pattern is present in the history of European art during the past six centuries.

Looking at the embedding, some other observations can be made. Along Dimension 1, there appears to be a transition of brushstroke coarseness, from fine brushstrokes on the left to coarse brushstrokes on the right side. Brushstroke coarseness can be one of the possible features describing the embedding. As Figure 6B suggests, the least coarse brushstrokes belong to the modern paintings, while the coarsest ones belong to the impressionists’ paintings from the 19th century. This trend in brushstroke coarseness can be one of the possible features describing the embedding and could have been used by participants as a way to differentiate styles. Another observation is a color gradient in the Dim1–Dim2 plane, from green apples on the top left, to yellow and red apples at the bottom. This gradient suggests that color could also have been used to differentiate styles. These two suggestions will be investigated in Experiment 2.

In summary, while the results clearly show a robust style space, we have yet to analyze it further. As we tentatively concluded, there appears to be a trend in Dimension 1 that relates to brushstroke coarseness and a trend in Dimension 2 related to hue, which might imply that Dimension 3 could be associated with color saturation and/or brightness. To quantify these latent trends, we conducted a second experiment where we used both perceptual attribute ratings and color measurements.

## Experiment 2: Explaining the embedding

Marković and Radonjić (2008) made a distinction between explicit and implicit features. Implicit features refer to subjective impressions (such as how pleasant a painting appears) while explicit features describe “physical properties” of the painting (such as form, color). We chose to define a number of explicit features that potentially contribute to style perception of the apple cut-outs from Experiment 1. Besides subjective rating data, we measured color statistics to account for the possible contribution of color.

### Method

#### Perceptual attributes

The nine visual attributes that we used were Glossiness, Smoothness, Three-dimensionality, Convincingness, Shadow contrast, Colorfulness, Brightness, Brushstroke coarseness, and Contrast between apple and background. Glossiness and Smoothness are typical object-specific features of apples while the other features refer more to how the apple has been depicted. Some of these have been used previously by, for example, Marković and Radonjić (2008), who used semantic differentials: Three-dimensionality as voluminosity–flat, Convincingness as realistic–abstract, Colorfulness as multicolored–unicolored, Brightness as light–dark, and Brushstroke coarseness as strong brushstrokes–soft brushstrokes. In addition, Convincingness (or realism in different terms) was used in several previous studies (Berlyne & Ogilvie, 1974; Ruth & Kolehmainen, 1974; O’Hare & Gordon, 1977; Chatterjee, Widick, Sternschein, Smith, & Bromberger, 2010); brushstroke coarseness (or clear–indefinite in different terms) was also used in several previous studies (Skager, Schultz, & Klein, 1966; Berlyne & Ogilvie, 1974; O’Hare, 1976; Hasenfus, Martindale, & Birnbaum, 1983; Chatterjee et al., 2010). As contrast was concluded to be connected with perceived glossiness (Marlow & Anderson, 2013; Di Cicco, Wijntjes, & Pont, 2019), we also included Shadow contrast and Contrast between apple and background in our study.

In the online experiment, each attribute scale was defined by two contrasting concepts, listed in Table 1 as left and right labels at either end of the continuous rating scale. No additional information was provided about the attributes to be assessed.

**Table 1.** Keywords of rating scales for attributes rating.

Attributes	Left label	Right label
Glossiness	matte	glossy
Smoothness	rough	smooth
Three-dimensionality	flat	three-dimensional
Convincingness	unrealistic	realistic
Shadow contrast	low	high
Colorfulness	monochrome	colorful
Brightness	dark	bright
Brushstroke coarseness	fine	coarse
Contrast between apple and background	low	high

#### Participants

In total, 224 unique participants recruited from AMT completed Experiment 2 (95.1% were from North America). Each of the nine attributes was rated by 30 unique participants, with 270 responses in total. Forty participants rated more than one attribute.

#### Stimuli and procedure

**Figure 7.**
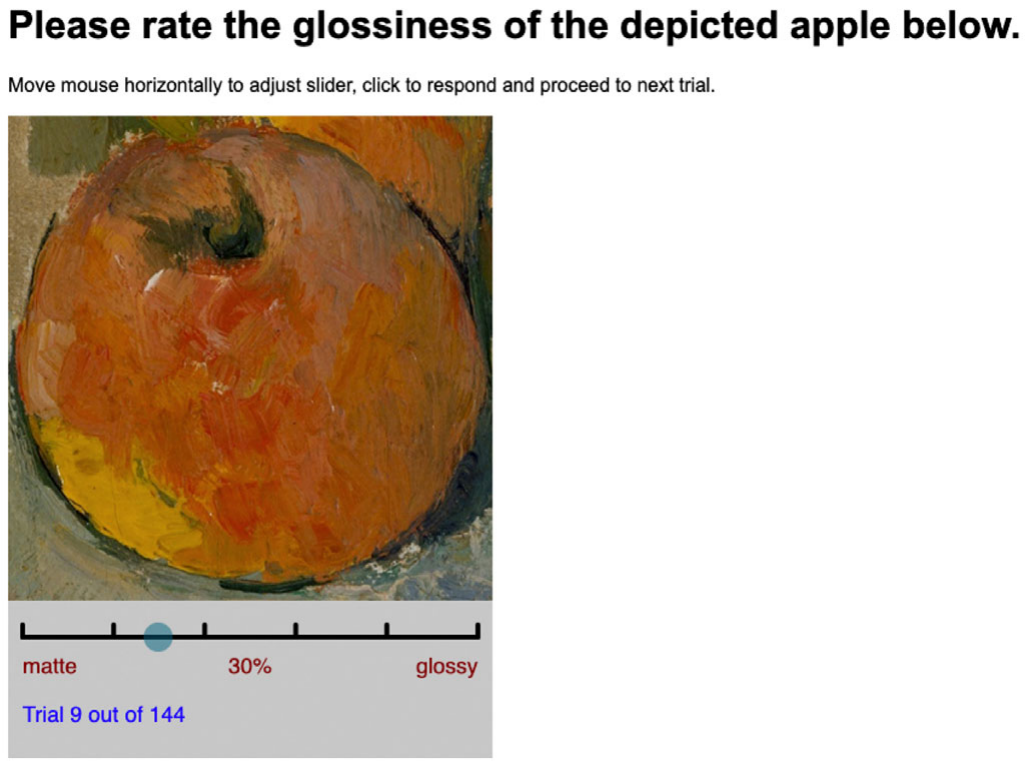
Experiment 2 interface for Glossiness. In each trial, participants were presented with a single cut-out. They could move the mouse horizontally to adjust the rating slider from matte to glossy. With a mouse click, they proceeded to the next trial.

The same 48 stimuli as in Experiment 1 were used in Experiment 2 (as shown in Figure 2). Before the actual experiment started, each participant would first read the consent form and instructions for the experiment. They could only proceed if they gave their consent by clicking “continue” after reading the consent form. Then they went through 15 practice trials, to familiarize with the interface and operation. One stimulus was displayed in each trial (as shown in Figure 7). Participants were asked to rate a certain attribute on a continuous scale with six markers and numerical feedback ranging between 0% and 100%. Each stimulus rating was repeated three times in a fully randomized set, resulting in 144 trials for each HIT.

#### Color measurements

In addition to the subjective ratings, we also computed color data from the apple images. To do so, we masked each apple image with a circular mask with a width of 75% of the image. In this way, colors almost certainly came from the apple and not from its surrounding. Colors were converted to CIELCh color space using the polar coordinates C* (chroma or relative saturation), Hue (hue angle), and L* (lightness). Chroma was defined as $\sqrt{a^{*2}+b^{*2}}$ and thus related to saturation, while Hue was defined by the hue angle, that is, tan ^−1^(*b*/*a*), values normalized between 0 and 1.

### Results

#### Rating agreement

For each attribute, we first performed validity checks based on average trial time and correlation with other participants. Data from participants who spent on average less than 1 second per trial were omitted (but were financially compensated). This threshold was based on similar experiments one of the authors conducted before (Van Zuijlen et al., 2020) and inspection of the time distribution in the current experiment. After the exclusion of the participants who spent less than 1 second, between 19 to 23 participants remained per attribute. After initial inspection, we found that a number of these participants seemed to misinterpret the polarity of the rating (i.e., had large but negative correlations with the group mean). Because the number of these cases could vary per attribute and thus result in unequal group sizes if we used “negative correlation” as a criterion, we decided to choose the top 15 participants.

**Figure 8.**
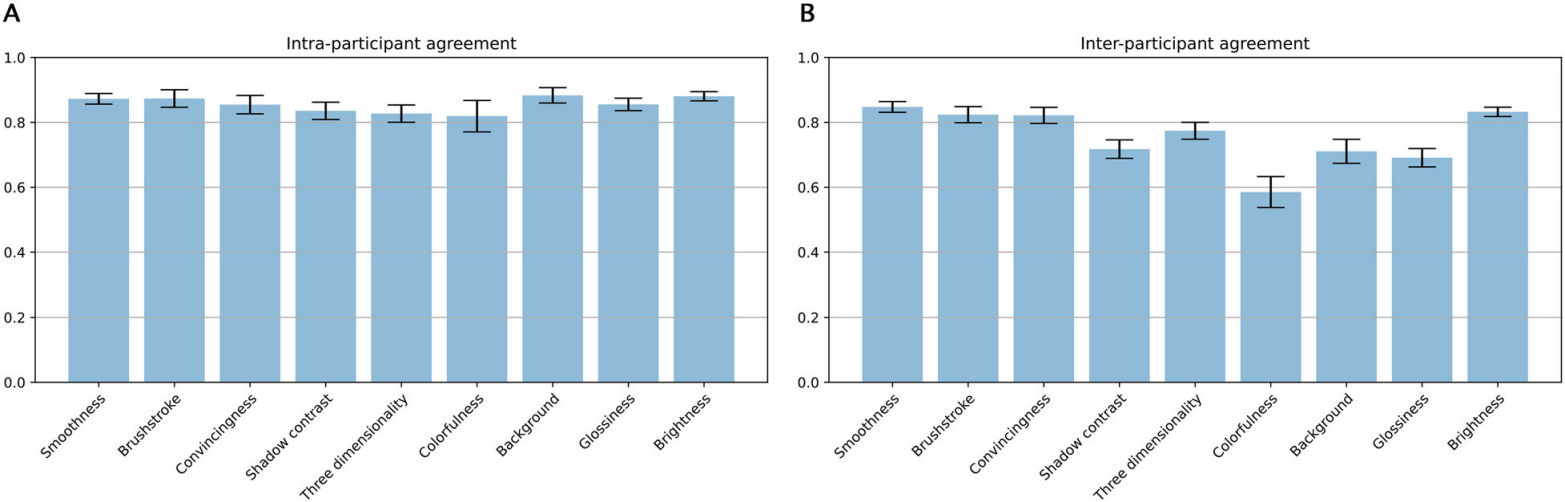
(A) Mean values and standard errors of correlations within three repetition measurements for 15 participants of each attribute. (B) Mean values and standard errors of correlation with a mean for 15 participants of each attribute.

As recommended by Martinez, Funk, and Todorov (2020), we first performed an intraparticipant reliability analysis before determining the interparticipant agreement. Figure 8A shows mean values and standard errors of correlations within three repeated measurements for each attribute.

As for interparticipant agreement, we first calculated the median rating over the three repetitions. We then correlated all the individual median ratings with the group mean (excluding the individual). Figure 8B shows mean values and standard errors of correlation with the mean for the participants of each attribute.

We found varying degrees of interparticipant agreement, which can be interpreted as perceptual ambiguities (high correlation, low ambiguity and vice versa). The interparticipant agreement varied between 0.85 and 0.59, with the highest scores for Smoothness, Brightness, Brushstroke, and Convincingness. The lowest score was for Colorfulness, with the others in between. The relatively constant high intrarater reliability correlations (all above 0.8) in Figure 8A suggest that differences between observers for the various attributes are truly due to interobserver ambiguities.

#### Multiple linear regression of perceptual attributes

**Figure 9.**
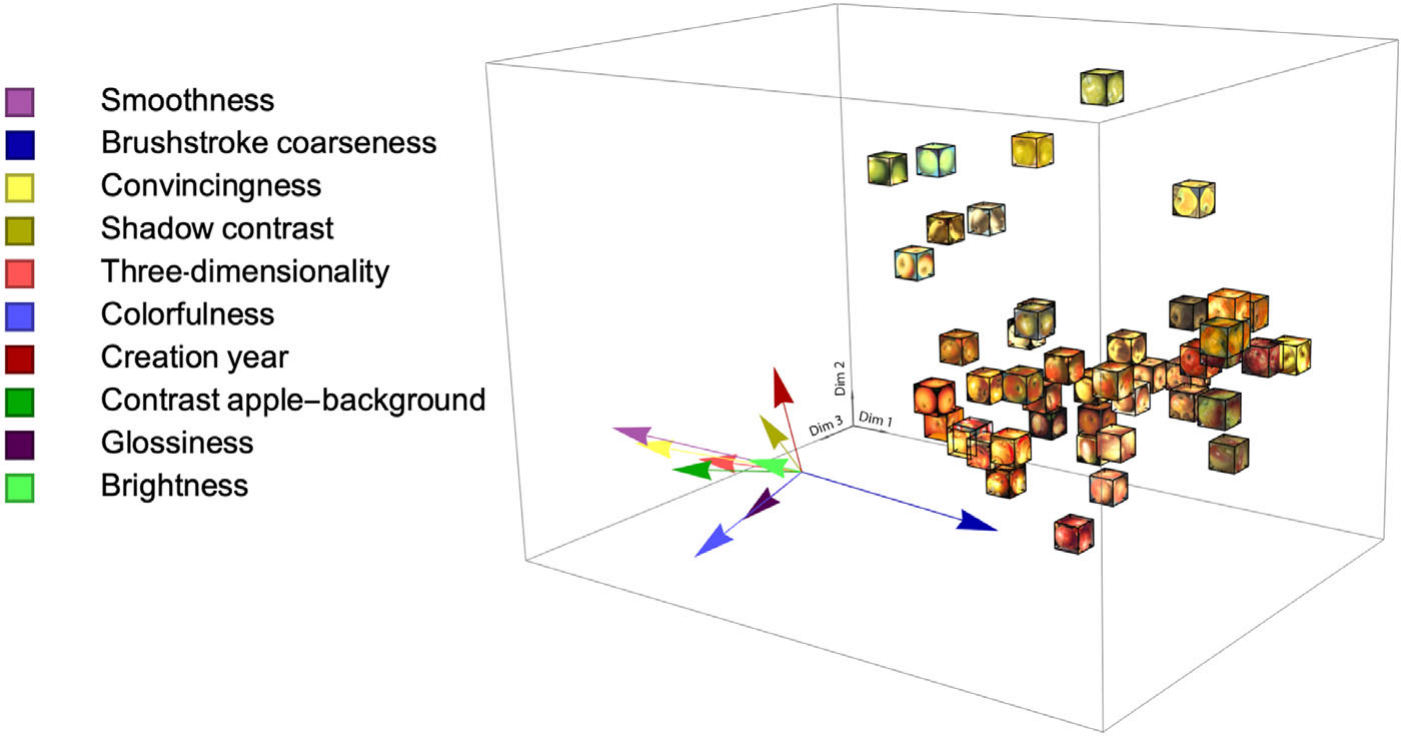
Three-dimensional MDS configuration embedded with nine attribute vectors and creation year vector.

Figure 9 presents the results of the multiple linear regressions within the MDS embedding from Experiment 1, using the three dimensions as independent variables and the attributes as dependent variables. The orientation of the vector indicates the direction that yielded the best regression, while the length indicates strength of the regression (*R*^2^). Table 2 denotes corresponding adjusted *R* square values, overall *p*-value, and weights (beta coefficients) plus *p*-values for each dimension of the fit per attribute. The following attributes have a high overall fit within the MDS embedding: Smoothness, Brushstroke coarseness, Convincingness, and Shadow contrast. The remaining attributes are moderately (Three-dimensionality, Colorfulness, Contrast between apple and background) or only weakly correlated (Glossiness, Brightness).

**Table 2.** Multiple linear regression of perceptual attributes.

	Adjusted	Overall						
	*R* square	*p*-value	Dim1	*p*-value	Dim2	*p*-value	Dim3	*p*-value
Smoothness	0.88	0.000^***^	−0.77	0.000^***^	0.08	0.145^NS^	0.17	0.031^*^
Brushstroke	0.88	0.000^***^	0.79	0.000^***^	−0.10	0.079^NS^	−0.12	0.167^NS^
Convincingness	0.85	0.000^***^	−0.64	0.000^***^	−0.05	0.919^NS^	0.06	0.409^NS^
Contrast	0.70	0.000^***^	−0.42	0.000^***^	0.02	0.709^NS^	−0.29	0.000^***^
3D	0.68	0.000^***^	−0.46	0.000^***^	−0.07	0.248^NS^	−0.08	0.388^NS^
Colorfulness	0.61	0.000^***^	−0.01	0.712^NS^	−0.16	0.002^**^	0.61	0.000^***^
Background	0.50	0.000^***^	−0.44	0.000^***^	0.00	0.996^NS^	0.25	0.045^*^
Glossiness	0.26	0.001^***^	−0.22	0.002^**^	−0.23	0.01^**^	0.21	0.104^NS^
Brightness	0.20	0.005^**^	−0.21	0.026^*^	0.19	0.101^NS^	0.43	0.014^*^

*Note*: Brushstroke: Brushstroke coarseness; Contrast: Shadow contrast; 3D: Three-dimensionality; Background: Contrast between apple and background.

* *p* < 0.05.

^**^ *p* < 0.01.

^***^ *p* < 0.001.

^NS^Means not significant (*p* > 0.05).

As Table 2 shows, all attributes except Colorfulness can be significantly associated with Dimension 1 from the Experiment 1 embedding, with Glossiness and Brightness only weakly associated. Dimension 2 has only weak associations with Colorfulness and Glossiness. Finally, Dimension 3 has a unique high association with Colorfulness and a moderate one with Brightness.

#### Fitting of color data

Table 3 shows per color coordinate (Hue, Chroma, Lightness) the adjusted *R*^2^ values, overall *p*-value, and weights plus *p*-values for each dimension of the MDS embedding from Experiment 1. Hue and Chroma both have significant overall fittings, while Lightness is not significantly associated with the 3D perceptual style space. Hue is primarily associated with Dimension 2 but also has some weight on Dimension 1. The only significant weight for Chroma is on Dimension 3. We also provide a correlation matrix of the nine attributes and three color measurements in the supplementary material.

**Table 3.** Multiple linear regression of color measurements.

	Adjusted	Overall						
	*R* square	*p*-value	Dim1	*p*-value	Dim2	*p*-value	Dim3	*p*-value
Hue	0.55	0.000^***^	−0.05	0.008^**^	0.16	0.000^***^	−0.05	0.103^NS^
Chroma	0.55	0.000^***^	0.02	0.528^NS^	−0.01	0.802^NS^	0.53	0.000^***^
Lightness	0.04	0.186^NS^	−0.07	0.194^NS^	0.11	0.115^NS^	0.09	0.401^NS^

*Note*:

** *p* < 0.01.

*** *p* < 0.001.

NS Means not significant (*p* > 0.05).

**Figure 10.**
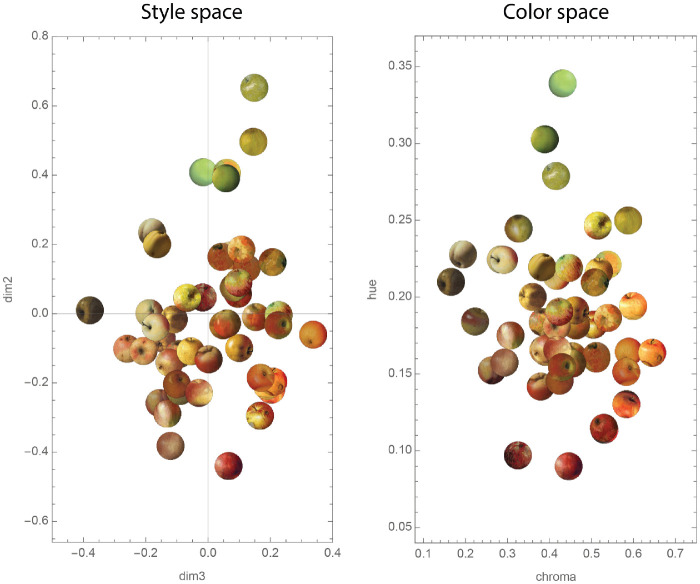
Comparison between Dim2–Dim3 plane of the MDS embedding (on the left) and 2D plane of Hue and Chroma measurements (on the right).

To illustrate the relation between image color coordinates Hue and Chroma and Dimensions 2 and 3 of the embedding, we plotted the Dim2–Dim3 projection of the embedding next to the Hue–Chroma plot. The result can be seen in Figure 10. A visual comparison between the style space and the color space underscores the strong association of Hue and Chroma with Dim2 and Dim3, respectively. It should be noted that although Hue has its primary weight on Dimension 2, the positive correlation between Hue and creation year (both highly correlated with Dimension 2) is low (*r* = 0.35), the two vectors of creation year and Hue having a substantial angle of 40.54 degrees because of the negative correlation between Hue and Dimension 1. In addition, the relation between creation year and Dim2 was explored further by calculating the partial correlation while controlling for Hue (*r* = 0.66; *p* < 0.001). The resulting correlation shows a small drop with respect to the original correlation (*r* = 0.69).

### Discussion

All visual attributes (highly to weakly) correlate significantly with the 3D embedding, as can be seen by the general adjusted *R*^2^ values in Table 2. The most prominent attributes are Smoothness, Brushstroke, and Convincingness, and the least contributing attributes are Brightness and Glossiness. For the color measurements, Hue and Chroma have significant overall correlations with the 3D embedding. Lightness has no significant correlation with the embedding, which is in line with the low contribution of Brightness to the perceptual attributes analysis. Finally, the best-fitting attributes are about spatial aspects of the paintings to which Convincingness is firmly associated, where Convincingness in its turn can be associated with realism according to O’Hare (1976).

Although all attributes (apart from Colorfulness and, to a lesser extent, Brightness) correlated significantly with Dimension 1, Brushstroke coarseness and Smoothness were the strongest ones. Similar findings were reported in previous studies (Berlyne, 1973; Klein, 1968; O’Hare & Gordon, 1977; Skager et al., 1966; O’Hare, 1976; Gardner, 1974), with similarly defined attribute names (e.g., clarity, texture). For instance, O’Hare (1976) reported in his study that the second dimension in his findings could be interpreted as clarity or clear definition of detail, from sharp outlines to diffuse and indefinite outlines. Gardner (1974) also reported that texture (brushstroke shapes, lightness gradients, etc.) makes a significant contribution to an artist’s style. Elgammal et al. (2018) reported a correlation between their second dimension and Wölfflin’s principle of linear versus painterly, which is connected to clarity of outline (brushstroke).

Dimension 2 corresponded strongly with creation year, as shown in Experiment 1. Interestingly, none of the attributes correlated with this dimension, except relatively weak negative correlations for Colorfulness and Glossiness. From Experiment 1, it was already visible that Hue could possibly also be related to Dimension 2. Indeed, the color statistics for Hue show a high coefficient of determination (*R*^2^ = 0.55), which originates from a direction mostly in the positive Dimension 2 direction (the significant weight of 0.16 in Table 3) and to a lesser degree in the negative Dimension 1 direction (the significant weight of −0.05 in Table 3). This becomes visually clear when again looking at Figure 5, where a clear transition from red to green is visible in Dimension 2, and one may also see more yellow/greenish apples on the left side than on the right side in Dimension 1. Although the trend is clearly visible, the interpretation is less straightforward, and we will continue this in the General discussion.

Dimension 3 is related to Colorfulness, another suggestion that participants might have used color information for the similarity judgments in our study. In addition, fitting the results from the color measurements suggested Dimension 3 was connected to Chroma only. It should be noted that the rating scale of Colorfulness was defined by monochrome to colorful, and hence it was expected that participants interpreted the term “colorfulness” as hue diversity, but, the results suggest that they interpreted the term more as saturation. In other words, semantic reasons might have caused different interpretations, which is also suggested by the lowest interparticipant correlation for Colorfulness (see Figure 8B, low correlation, high ambiguity).

In the attribute rating experiment, relatively high interparticipant correlations were found (Figure 8B), which is in line with the significant intersubject consistency reported by Berlyne and Ogilvie (1974). Next to Brightness, the lowest agreement has been found for Shadow contrast, Contrast between apple and background, and Glossiness. While this could partially be semantic, it may also be visual. Especially since Glossiness is a term that is generally unambiguous, the relatively low agreement score could therefore indicate that there is not much variation in Glossiness within the 48 apples. Three-dimensionality scored higher, followed by Smoothness, Brushstroke coarseness, and Brightness.

## General discussion

We have measured the perception of style using a supposedly constant motif, the apple, by using square cut-outs of paintings. Gombrich’s (1968) description of style (“Style is a distinctive, and therefore recognizable”) was operationalized in two experiments: the first quantifying distinction by performing a landmark MDS experiment, the second describing the resulting embedding, which can be related to recognizing style. The results reveal an interesting, nonrandom multidimensional embedding of 48 apple depictions that are related through various visual features. The embedding is even more interesting considering only low-level information was left in the square cut-outs. Previous studies (Wallraven et al., 2009; Siefkes & Arielli, 2018) believed humans need high-level information to perceive different styles, which was removed as much as possible in our study. It suggests that low-level information might be sufficient for participants to perceive style differences.

In Experiment 1, we also found a strong correlation between creation year and our perceptual space, with both a linear fit along Dimension 2 (*r* = 0.69) and a circular fit in the Dim1–Dim2 plane (*r* = 0.70). These correlations were surprising, considering all the high-level and mid-level information; in other words, all the time-related items and surroundings (e.g., clothes, house interior) that can provide information about creation time were removed. Indeed, connections between the perceptual space and paintings’ creation year have been reported in other studies, but all with the whole paintings as stimuli. Berlyne and Ogilvie (1974) found a high multiple correlation (>0.8) between their 3D perceptual space and artists’ year of birth, which roughly scales with the creation year of the paintings. And Berlyne and Ogilvie (1974) interpreted the first dimension in their perceptual space as old versus modern. Elgammal et al. (2018) also found a temporal pattern while using computational methods instead of human judgments. Their embedding was achieved by training neural networks on WikiArt-style labels. Thus, similar findings from both human judgment and computer algorithm indicate a relatively robust correlation between style and time. And if we consider the circular fit, the creation year changes in a cycle of both texture and Hue.

In Experiment 2, we described the 3D perceptual style space with multiple linear regressions of nine attributes and color measurements. The first dimension of the style embedding clearly related to many of the attributes, most prominently Smoothness and Brushstroke coarseness, but also others like Convincingness, Shadow contrast, and Three-dimensionality. Except Convincingness being a higher-level attribute, all other mentioned attributes are related to spatial properties. As shown in Figure 9 and Table 2, Smoothness, Shadow contrast, Three-dimensionality, and Convincingness all point in the same direction, which indicates that increasing Smoothness, Shadow Contrast, and Three-dimensionality could enhance Convincingness. Similar positive correlations between Contrast, Three-dimensionality, and Convincingness was reported in a previous study (Di Cicco et al., 2019). Smoothness and Brushstroke coarseness have opposite directions in the 3D embedding, implying they have an almost perfect negative correlation, which appears logical as they indeed seem semantic opposites. But it should be noted that the instructions for the Smoothness rating experiment explicitly mentioned the apple skin with the intention that Smoothness should relate to what is represented (the apple) while Brushstroke coarseness clearly relates to the medium. However, our results pointed at a transfer between these two modes, perhaps because apples painted in a rough manner cannot easily be judged as being smooth. Such a phenomenon can be further tested in controlled experiments where motif and medium are systematically varied.

The remaining two dimensions are associated with color. The second dimension is associated with Hue and the third dimension with Chroma as well as the attribute Colorfulness. It seems there is some connection between Hue and creation year since they are both positively correlated to Dimension 2. Indeed, from the beginning of the 19th century, the production of new synthetic pigments exploded, leading to a variety of colors unheard of in earlier centuries (Ball, 2003; Wilson-Bareau, 1991). Artists such as Rembrandt had to make do with about a dozen pigments, while Monet or Van Gogh could literally choose hundreds of different pigments. This has led to an increase of saturation of violets and greens, for example. Another possibility is that it shows the history of the painted objects, in our case apples. Despite their seemingly independence of historical developments in fashion or the development of technology, it is quite possible that European cultivated apples have a history of their own, in which there has been a gradual increase in saturated green varieties over the past century or so. However, even if we only consider the linear fit of the creation year with Dimension 2, this time dimension still cannot be fully explained by Hue change, given the creation year and Dimension 2 have a high correlation (*r* = 0.69), while the creation year and Hue have a low correlation (*r* = 0.35), and the two vectors of the creation year and Hue have a substantial angle of 40.54 degrees between them. This conclusion is convincingly supported by the fact that the partial correlation between Dimension 2 and creation year, controlling for Hue, has a value of 0.66, being close to the original correlation.

Although color measurements could not explain the time dimension, the contribution of color in style perception was robust and also reported in early studies. Gardner (1974), for example, concluded that both color and texture played a significant role in style detection. Interestingly, Dimension 1 in our study is mainly associated with spatial attributes, such as brushstroke coarseness, smoothness, shadow contrast, and three-dimensionality, which can also be interpreted as texture. Hence, we reached the same conclusion as Gardner (1974) that texture and color are two important variables for pictorial style perception.

In this study, we showed that in judging matters of style, participants in our two experiments demonstrated high intersubjective agreement, in line with earlier studies on the perception of style in art. We also found that participants by and large followed the historical timeline when performing their matching tasks. In our case, this concerned only small details of sometimes much larger paintings (our apple stimuli), thus removing such important aspects as composition, mood, or general intention of the work of art as a whole. With regard to paintings, people are apparently quite capable of looking at the “how” of a painted subject. They show a definite sense of style.

Experiment 2 showed some of the perceptual ingredients on which this sense of style may rely, but there did not seem to be a single, one-dimensional perceptual factor explaining the results. Perhaps our sense of pictorial style is just one member of a much larger family of human sensitivities for the “how” of something made or done by other humans: for example, handwriting styles, dialects, speech habit, and dancing styles (Hasenfus et al., 1983). In all such activities, people detect various components simultaneously, as if they were Gestalts. Further research on recurring motifs in the history of art (e.g., hands, textile folds) may get us closer to discovering the various roots of this important sense of style in humans.

## Supplementary Material

Supplement 1
