# Supplementary figures and images for: Zooming in on style: Exploring style perception using details of paintings
Source: J Vis. 2023 Jun 2;23(6):2. doi: 10.1167/jov.23.6.2 (PMC10243498; doi:10.1167/jov.23.6.2)

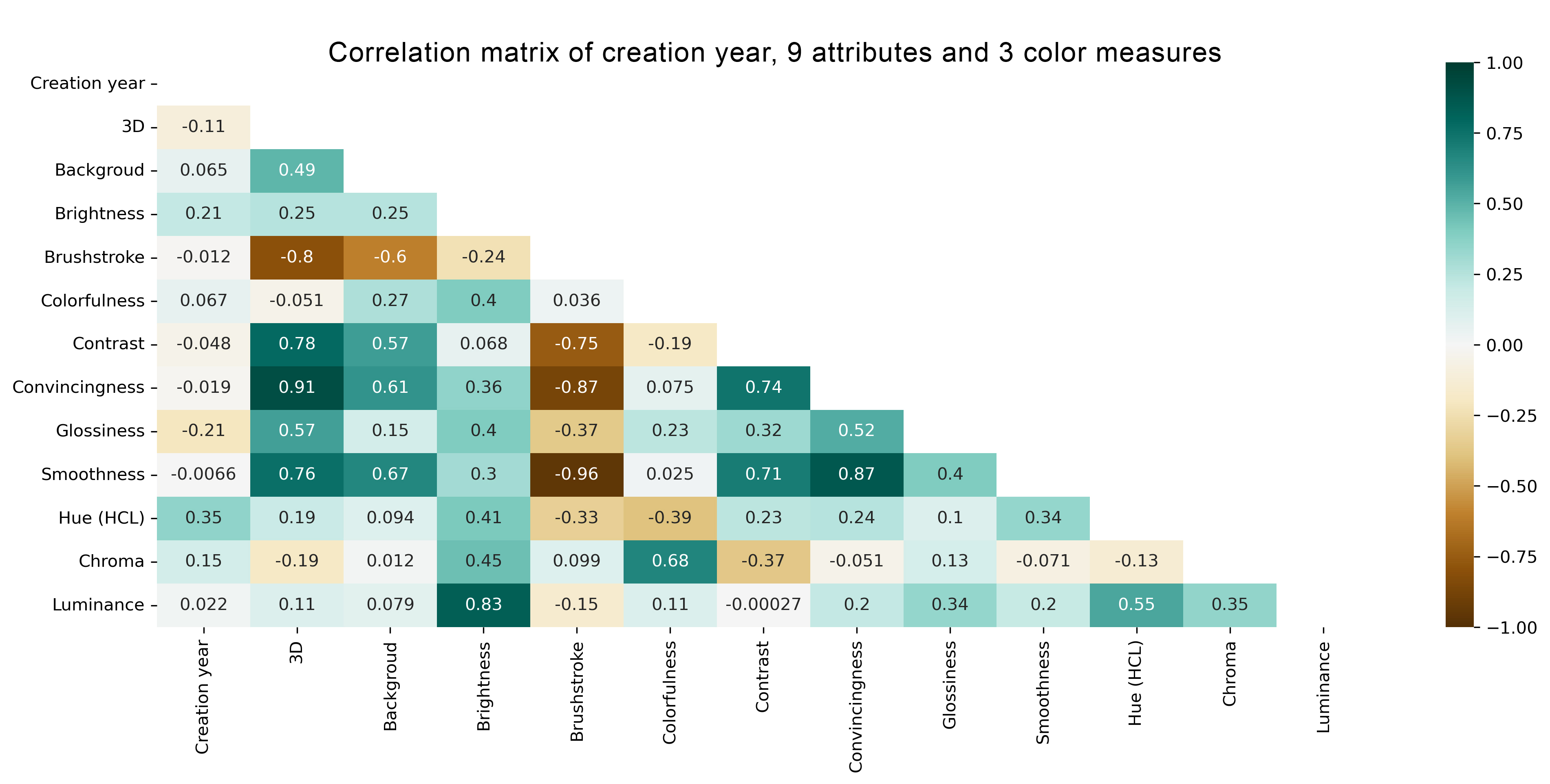

Supplement: Supplement 1 [file jovi-23-6-2_s001.jpg]
